# Active and Healthy Ageing and Independent Living 2016

**DOI:** 10.1155/2016/8062079

**Published:** 2016-10-12

**Authors:** Maddalena Illario, Miriam M. R. Vollenbroek-Hutten, D. William Molloy, Enrica Menditto, Guido Iaccarino, Patrik Eklund

**Affiliations:** ^1^Department of Translational Medical Sciences, Federico II University and R&D, Federico II University Hospital, Naples, Italy; ^2^Roessingh Research and Development, Telemedicine Group, Enschede, Netherlands; ^3^Telemedicine Group, Faculty of Electrical Engineering, Mathematics and Computer Science, University of Twente, Enschede, Netherlands; ^4^Centre for Gerontology and Rehabilitation, St. Finbarr's Hospital, University College Cork, Cork, Ireland; ^5^CIRFF, Center of Pharmacoeconomics, Federico II University, Naples, Italy; ^6^Department of Medicine and Surgery, University of Salerno, Salerno, Italy; ^7^Department of Computing Science, Umeå University, Umea, Sweden

Population ageing is a global trend linked to the progressive improvement of living conditions and to the progress of the medical fields [1]. Sustainability issues related to the provision of social and health services are emerging in developed countries [2] that are implementing a number of strategies to ensure good quality of life for all ages and older adults, focusing on maintaining independence and ensuring an active life as people age in their own environment [3, 4]. Several dimensions have traditionally been linked to the management of health in older adults that were mostly related to physical functionality. Currently, an emerging role is being identified for additional factors that overcome the boundaries of health but nonetheless influence health outcomes, such as lifestyles, built environment, and social inclusion [5, 6]. This special issue provides examples of innovative, cross-sectorial strategies that contribute directly or indirectly to improving the quality of life for older adults and their closer ones, making our health and social care systems more efficient and sustainable.

The need to provide tailored models to be implemented on a large scale is addressed by Md. N. Haque, whose study is aimed at providing evidences for prioritizing the policy agenda in Thailand. The author provides an overview of active ageing level and its discrepancy in different regions (Bangkok, Central, North, Northeast, and South) of Thailand has been examined for prioritizing policy agenda to be implemented. In his paper, Haque makes an attempt to test preliminary active ageing models for Thai older persons by evaluating the active ageing index (AAI ranges from 0 to 1) and using nationally representative data and confirmatory factor analysis approach. The study results show that active ageing level of Thai older persons is not high (mean AAI for female and male older persons are 0.64 and 0.61, respectively, and those are significantly different (*p* < 0.001)). Mean AAI in Central region is lower than those in North, Northeast, and South regions but there is no significant difference in the latter three regions of Thailand. The author urges a special emphasis to the Central region and to the need for a central policy aimed at increasing active ageing level. The study suggests that the implementation of an Integrated Active Ageing Package (IAAP), containing policies for older persons to improve their health and economic security, to promote participation in social groups and longer working lives, and to arrange learning programs, would be helpful for increasing older persons' active ageing level in Thailand.

The link between social inclusion and health is addressed by C. McKibbin et al. and by A. Rapacciuolo et al. in two different locoregional contexts: rural communities and metropolitan areas.

C. McKibbin et al. investigate how health status and social networks are associated with resilience among older adults and contribute to their ability to remain in rural and remote communities as they age. The authors examined the association of health status and social networks with resilience among older adults dwelling in a rural and remote county in the Western United States. A random sample of 198 registered voters aged 65 years or older from a frontier Wyoming county was selected for the study. Hierarchical linear regression was used to examine the association of health status and social networks with resilience. Health status was also examined as a moderator of the relationship between social networks and resilience. The results show that family networks (*p* = 0.024) and mental health status (*p* < 0.001) significantly predict resilience. Mental health status moderated the relationship of family (*p* = 0.004) and friend (*p* = 0.021) networks with resilience. Smaller family and friend networks were associated with greater resilience when mental health status was low but not high. The authors conclude that efforts to increase mental health status may improve resilience among older adults in rural environments, particularly for those with smaller family and friends networks.

Complementary to the previous study, A. Rapacciuolo et al. describe the impact of social and cultural engagement and dieting on well-being and resilience in a group of residents in the metropolitan area. They highlight that a number of related isolation factors, inadequate transportation system and restrictions in individuals' life-space, have been associated with malnutrition in older adults. Since eating is a social event, isolation can have a negative effect on nutrition. Cultural involvement and participation in interactive activities are essential tools to fight social isolation and they can counteract the detrimental effects of social isolation on health. They developed an ad hoc questionnaire to investigate the relationship between cultural participation and well-being and resilience in a sample of residents in the metropolitan area of Naples. The questionnaire includes a question on adherence to diet or to a special nutritional regimen; in addition, it is required to refer to height and weight. We investigated the relationship between BMI, adherence to diet, and perceived well-being (PWB) and resilience in a sample of 571 subjects over 60 years of age. In the paper, the authors present evidence that engagement into social and cultural activities is associated with higher well-being and resilience, in particular in females over 60 years of age.

Physical activity is a key component of healthy lifestyles: M. J. Turner et al., C. A. Parker and R. Ellis, and R. C. Mason et al. describe different experiences to approach this issue in older adults.

M. J. Turner et al. assess the influence of participating in regularly scheduled activity on weekly physical activity levels in an independent-living older adult population and investigate lifetime exercise history and sex differences, in an effort to understand how they relate to current weekly step activity. Total weekly step counts, measured with a pedometer, were assessed in two older adult groups: the first consisted of members of a local senior center who regularly used the fitness facility (74.5 ± 6.0 yrs; mean ± SD), while the second group consisted of members who did not use the fitness facility (74.8 ± 6.0 yrs). Participants also completed the Lifetime Physical Activity Questionnaire (LPAQ). The LPAQ suggested a significant decline in activity with ageing (*p* = 0.01) but no difference between groups (*p* = 0.54) or sexes (*p* = 0.80). A relationship was observed between current step activity and MET expenditure over the past year (*p* = 0.008, *r* ^2^ = 0.153) and from ages of 35–50 years (*p* = 0.037, *r* ^2^ = 0.097). The lack of difference in weekly physical activity level between our groups suggests that independent older adults will seek out and perform their desired activity, either in a scheduled exercise program or through other leisure-time activities. Also, the best predictor of current physical activity level in independent-living older adults was the activity performed over the past year.

C. A. Parker and R. Ellis investigated whether electronic messaging would increase aerobic physical activity (PA) among older adults. Participants were active older adults (*n* = 28; M age = 60 years, SD = 5.99, and range = 51–74 years). Using an incomplete within-subjects crossover design, participants were randomly assigned to begin the 4-week study receiving the treatment condition (a morning and an evening text message) or the control condition (an evening text message). Participants self-reported min of completed aerobic PA by cell phone text. The 1-way within-subjects ANOVA showed significant group differences (*p* < 0.05). Specifically, when participants were in the treatment condition, they reported significantly greater average weekly min of aerobic PA (M = 96.88 min; SD = 62.9) compared to when they completed the control condition (M = 71.68 min; SD = 40.98). They conclude that electronic messaging delivered via cell phones was effective at increasing min of aerobic PA among older adults.

R. C. Mason et al. investigated the effects of exercise on the physical fitness of high and moderate-low functioning older adult women. The primary purpose of their study was to observe the exercise habits of older adults with different levels of physical function to determine any differential effects of exercise on their physical fitness and functional ability. The effects of 10 weeks of community-based exercise on the cardiovascular endurance, muscular strength, flexibility, and balance fitness components of older adult women with high and moderate-low levels of physical function were evaluated. This study provides several noteworthy findings. Firstly, it shows that community-based exercise programs offering a variety of exercise types to people with varying levels of functional ability can be useful in maintaining or improving fitness and independence. Secondly, it suggests that such programs may also be capable of improving the self-efficacy of lower functioning older adults toward performing daily tasks. Additionally, self-report instruments such as activity logs may be useful to track and gain an understanding of the exercise habits of older adults.

Research has demonstrated that active and healthy ageing can be enhanced by enabling societal infrastructure, urban planning, architecture of healthcare facilities, and personal accommodations throughout the life span. Yet, there is a paucity of research on how to bring together the various disciplines involved in a multidomain synergistic collaboration to create new living environments for ageing throughout the life span. E. Chrysikou et al.'s study aims to explore the key domains of skills and knowledge to consider in order to generate a conceptual prototype where healthcare professionals, architects, planners, and entrepreneurs may establish shared theoretical and experiential knowledge base, vocabulary, and implementation strategies, to create age-friendly living communities, that are fit also for persons with disabilities and chronic disease. The study focuses on the domains of knowledge that need to be included in establishing a learning model that focuses on the impact of the benefits toward active and healthy ageing, where architects, urban planners, clinicians, and healthcare facility managers are educated toward a synergistic approach at the operational level. A number of studies support the concept that health and well-being in later life are heavily influenced by behavioural factors and social conditions [7–9], and interventions targeting multiple behavioural and social factors are showing their effectiveness in promoting health and well-being during ageing [10–13]. Many governments are introducing policies to support effective lifestyle interventions to enhance active and healthy ageing and reduce the burden of age-related disease [14, 15]. To this purpose, using a one-size-fits-all approach demonstrated limited long-term effectiveness and posed the risk of generating on the contrary health inequalities. Integrating the design of the different interventions with subsets of indicators that are specific to the different settings (locoregional, rural, metropolitan, etc.) and with novel ICT tools will contribute to convenience, facilitate scalability, and allow personalisation to stratified target populations [16, 17].

*Maddalena Illario* *Maddalena Illario* *Miriam M. R. Vollenbroek-Hutten* *Miriam M. R. Vollenbroek-Hutten* *D. William Molloy* *D. William Molloy* *Enrica Menditto* *Enrica Menditto* *Guido Iaccarino* *Guido Iaccarino* *Patrik Eklund* *Patrik Eklund*
